# Supplementary material for: Intra-striatal AAV2.retro administration leads to extensive retrograde transport in the rhesus macaque brain: implications for disease modeling and therapeutic development
Source: Sci Rep. 2020 Apr 24;10:6970. doi: 10.1038/s41598-020-63559-7 (PMC7181773; doi:10.1038/s41598-020-63559-7)
Supplement: Supplementary file 1 — Supplementary Information. [file 41598_2020_63559_MOESM1_ESM.pdf]

SUPPLEMENTARY INFORMATION

Intra-striatal AAV2.retro administration leads to extensive retrograde transport in the rhesus macaque brain: implications for disease modeling and therapeutic development

Alison R. Weiss[1], William A. Liguore[1], Jacqueline S. Domire[1], Dana Button[1] and Jodi L. McBride[1, 2, 3*]

[1]Division of Neuroscience, Oregon National Primate Research Center, Beaverton, OR; Departments of Behavioral Neuroscience[2] and Neurology[3], Oregon Health and Science University, Portland, OR

Supplemental Table S1.

**AAV2.retro**									
	Caudate	Putamen	ACC	AMY	DPFC	DPMC	PMC	SSC	THALAMUS
Caudate		0.0003	0.0051	0.0011	0.0006	0.0009	0.0003	0.0002	0.0009
Putamen	0.0003		<0.0001	<0.0001	<0.0001	<0.0001	<0.0001	<0.0001	<0.0001
ACC	0.0051	<0.0001		1.0000	1.0000	1.0000	0.6605	0.2904	1.0000
AMY	0.0011	<0.0001	1.0000		1.0000	1.0000	1.0000	1.0000	1.0000
DPFC	0.0006	<0.0001	1.0000	1.0000		1.0000	1.0000	1.0000	1.0000
DPMC	0.0009	<0.0001	1.0000	1.0000	1.0000		1.0000	1.0000	1.0000
PMC	0.0003	<0.0001	0.6605	1.0000	1.0000	1.0000		1.0000	1.0000
SSC	0.0002	<0.0001	0.2904	1.0000	1.0000	1.0000	1.0000		1.0000
THALAMUS	0.0009	<0.0001	1.0000	1.0000	1.0000	1.0000	1.0000	1.0000	

**AAV2**									
	Caudate	Putamen	ACC	AMY	DPFC	DPMC	PMC	SSC	THALAMUS
Caudate		0.0012	0.0141	0.0140	0.0140	0.0139	0.0141	0.0139	0.0141
Putamen	0.0012		<0.0001	<0.0001	<0.0001	<0.0001	<0.0001	<0.0001	<0.0001
ACC	0.0141	<0.0001		1.0000	1.0000	1.0000	1.0000	1.0000	1.0000
AMY	0.0140	<0.0001	1.0000		1.0000	1.0000	1.0000	1.0000	1.0000
DPFC	0.0140	<0.0001	1.0000	1.0000		1.0000	1.0000	1.0000	1.0000
DPMC	0.0139	<0.0001	1.0000	1.0000	1.0000		1.0000	1.0000	1.0000
PMC	0.0141	<0.0001	1.0000	1.0000	1.0000	1.0000		1.0000	1.0000
SSC	0.0139	<0.0001	1.0000	1.0000	1.0000	1.0000	1.0000		1.0000
THALAMUS	0.0141	<0.0001	1.0000	1.0000	1.0000	1.0000	1.0000	1.0000	

Supplemental Table S1. Matrix of p-values from post-hoc comparisons of cell counts between brain regions. Tables report the p-values from one-tailed independent sample t-tests to compare the numbers of GFP+ cells in each ROI separately for each of the two serotypes, AAV2.retro (upper panel) and AAV2 (lower panel).

Supplemental Table S2.

High	Medium	Low	None
Dorsal prefrontal cortex	Somatosensory cortex	Parietal cortex	Occipital cortex
Ventral prefrontal cortex	Orbitofrontal cortex	Superior temporal cortex	Cerebellum
Dorsal premotor cortex	Rhinal cortex	Thalamus (MD/LD)	Thalamus (RE)
Ventral premotor cortex	Thalamus (LP/VPL)	Inferior temporal cortex	Thalamus (VA/VL)
Anterior cingulate cortex	Amygdala (BLN)	Substantia nigra, pars compacta	Substantia nigra, pars reticulata
Subthalamic nucleus	Claustrum	Globus Pallidus, int. segment	
Pre-supplemental motor cortex	Globus Pallidus, ext. segment		
Supplemental motor cortex			
Primary motor cortex			
Insular Cortex			

Supplemental Table S2. Relative levels of brain-wide mutant HTT expression following intra-caudate and intra-putamen injection of AAV2.retro-HTT85Q. Brain regions were qualitatively ranked relative to one another, considering cell number and density, and placed into categories of high, medium, low and minimal/none. All regions listed here had less expression compared to the injected regions of the caudate and putamen. MD- medial dorsal, LD- lateral dorsal, RE- nucleus reuniens, BLN- basolateral nucleus, int.- internal, ext.- external, VA- ventral anterior, VL- ventral lateral, LP- lateral posterior, VPL- ventral posterolateral.
